# Dysregulation of adenosine kinase isoforms in breast cancer

**DOI:** 10.18632/oncotarget.27364

**Published:** 2019-12-31

**Authors:** Bahar Shamloo, Nandita Kumar, Randall H. Owen, Jesica Reemmer, John Ost, R. Serene Perkins, Hai-Ying Shen

**Affiliations:** ^1^Department of Translational Neuroscience, Legacy Research Institute, Legacy Health, Portland, OR 97232, USA; ^2^Legacy Tumor Bank, Legacy Research Institute, Legacy Health, Portland, OR 97232, USA; ^3^Mid-Columbia Medical Center, The Dalles, OR 97058, USA

**Keywords:** adenosine kinase, breast cancer, adenosine kinase isoforms, metastasis, proliferation

## Abstract

Dysregulated adenosine signaling pathway has been evidenced in the pathogenesis of breast cancer. However, the role of adenosine kinase (ADK) in tumorigenesis remains unclear while it crucially regulates the removal and availability of adenosine. ADK has two isoforms that localize to discrete subcellular spaces: i.e., nuclear, long-isoform (ADK-L) and cytosolic, short-isoform (ADK-S). We hypothesized that these two ADK isoforms would be differentially expressed in breast cancer and may contribute to divergent cellular actions in cancer. In this study, we examined the expression profiles of ADK isoforms in breast cancer tissues from 46 patient and followed up with an *in vitro* investigation by knocking down the expression of ADK-L or ADK-S using CRISPR gene editing to evaluate the role of ADK isoform in cancer progression and metastasis of cultured triple-negative breast cancer cell line MDA-MB-231. We demonstrated that *(i)* ADK-L expression level was significantly increased in breast cancer tissues versus paired normal tissues adjacent to tumor, whereas the ADK-S expression levels were not significantly different between cancerous and normal tissues; *(ii)* CRISPR/Cas9-mediated downregulation of ADK isoforms, led to suppressed cellular proliferation, division, and migration of cultured breast cancer cells; *(iii)* ADK-L knockdown significantly upregulated gene expression of matrix metalloproteinase (ADAM23, 9.93-fold; MMP9, 24.58-fold) and downregulated expression of cyclin D2 (CCND2, -30.76-fold), adhesive glycoprotein THBS1 (-8.28-fold), and cystatin E/M (CST6, -16.32-fold). Our findings suggest a potential role of ADK-L in mitogenesis, tumorigenesis, and tumor-associated tissue remodeling and invasion; and the manipulation of ADK-L holds promise as a therapeutic strategy for aggressive breast cancer.

## INTRODUCTION

Adenosine plays a crucial role in metabolic regulation and many essential physiological functions in humans, such as vasodilation, immune response, inflammation, neuroprotection, arousal, and sleep [1]. Extracellular adenosine can accumulate in the microenvironment of cancerous tissue, leading to immunosuppression [2] and angiogenesis [3] – two common characteristics of cancer. While the dysregulation of adenosine and its signaling pathway has been evidenced in cancer [4–8], the blockade of adenosine signaling promotes antitumor responses [8]. Previous work on the adenosine signaling pathway demonstrated adenosine metabolic enzymes and adenosine receptors are tightly linked to the pathogenesis of breast cancer [9]. Adenosine A_2B_ receptor was identified as a target of the metastasis-inducing transcription factor FOS-related antigen 1 (FRA1) in triple negative breast cancer (TNBC) [10], and adenosine A_2A_ receptor activation promotes proliferation of breast carcinoma [11]. While adenosine changes in the microenvironment of cancerous tissue determines local activities of adenosinergic pathways, due to its rapid clearance and short half-life in the body, a better therapeutic approach may be to manipulate adenosine metabolism.

Metabolic removal of adenosine occurs either through its deamination by adenosine deaminase (ADA) to be converted to inosine or via phosphorylation by adenosine kinase (ADK) to form adenosine monophosphate [12, 13]. As ADK plays a major role in adenosine removal and its availability for downstream effects, this adenosine-ADK balance is strictly regulated in healthy cells [14]. ADK has two isoforms: a long isoform, ADK-L, dominantly located in the nucleus [15]; and a short isoform, ADK-S, located in the cytoplasm [16]. The expression of ADK-L and ADK-S is dynamic during development with differentiated patterning; for example, ADK-L is dominantly expressed in early postnatal brain development, but then an ADK-S expression pattern becomes dominant in adult brain tissue [17]. This differential expression suggests a distinctive role for ADK-L (versus ADK-S) on proliferation and differentiation – two major nuclear activities that are tightly linked to cancer pathology [18]. ADK-L is reported to affect epigenetic remodeling [19, 20] and is thought to preferentially function as a regulator of methyltransferase action through clearance of adenosine [17, 21]. On the other hand, ADK-S regulates extracellular adenosine concentration to activate various adenosine receptor subtypes and affects angiogenesis [22], inflammation [20], and immune suppression [23]. These functions of ADK isoforms strongly support a differential role for ADK-L versus ADK-S in cancer.

ADK expression is dysregulated in various cancer tissues [5, 21, 24], however, there has been no focused study on dissecting the roles of ADK isoforms in cancer cells. Particularly, the ADK-L mediated function and underlying mechanisms are still less known. Due to the distinct subcellular compartments, varying expression levels across different cancers, and differential actions on cellular biology between the two ADK isoforms, we hypothesized that ADK isoforms may have distinct effects in cancer pathology and, as such, specifically targeting ADK-L or ADK-S may provide a novel therapeutic approach for cancer treatment. New insights and approaches are necessary, particularly for those aggressive cancers that lack therapeutic targets, such as triple-negative breast cancer that does not respond to either hormonal therapies or Her2-targeted therapies. Therefore, in this study, we first characterized the expression profiles of ADK isoforms in different types of breast cancer. We compared the expression levels of ADK-L and ADK-S between the tumor and normal adjacent tissue (NAT) to the tumor of patients. In addition, to identify possible divergent roles of ADK isoforms, we conducted a follow-up *in vitro* study using our established CRISPR/Cas9 gene-editing approach to knockdown ADK-L or ADK-S isoforms in cultured MDA-MB-231 cell lines, and further evaluated the effects of manipulating each ADK isoform on the cell growth, viability, migration, and invasion ability of cultured breast cancer cells.

## RESULTS

### Disrupted expression profiles of ADK isoforms in breast cancer

**Figure 1:**
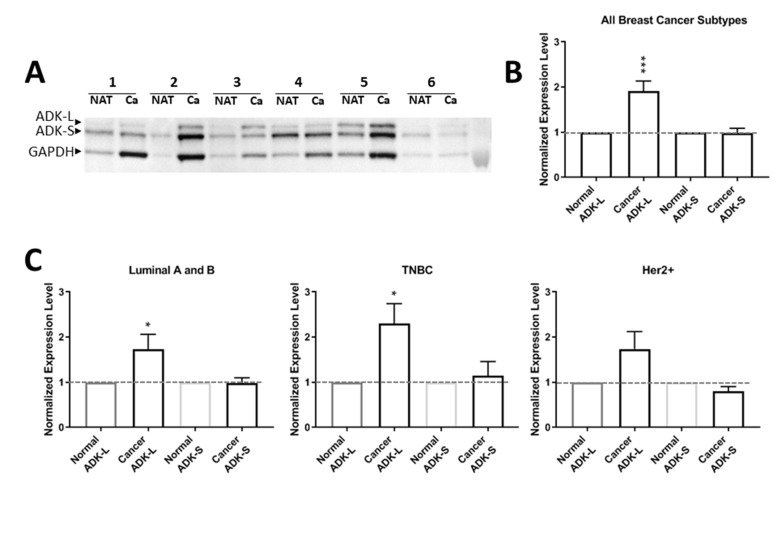
Expression profiles of ADK isoforms in cancer tumor versus NAT from 46 patients. **(A)** Representative Western blot of 6 TNBC patients’ cancer tissue (Ca) versus paired normal adjacent tissue of tumor (NAT). **(B)** Quantitative analysis of ADK-L and ADK-S expressions of pooled subtypes of breast cancer (a total of 46 patients): Luminal A and B positive, Her2+, and TNBC subtypes. **(C)** Quantitative analysis of ADK-L and ADK-S expression in 19 luminal A and luminal B positive subtype patients (left panel), 14 TNBC subtype patient (middle panel); and 13 Her2+ subtype patients (right panel). * p<0.05; *** p<0.001; red line indicates normalized 1-fold expression level of ADK in NATs.

To investigate the profile of ADK isoforms in breast cancer, we compared the expression levels of ADK-L and ADK-S in cancer tissues versus NAT controls in patients with breast cancer (n=46; Figure 1). To compare the expression profile of ADK isoforms in different patients, we normalized the expression level of ADK-S or ADK-L isoforms in cancer tissue from each patient to the corresponding paired NAT in the same patient; our Western blot data showed that expression of ADK-L significantly increased in breast cancer versus NAT controls (paired t-test, *t*=4.153, *df*=43, *p*=0.0002) (Figure 1A, 1B). Further analysis with separated subtypes of breast cancer revealed that ADK-L expression levels were significantly increased in both luminal A and B positive subtypes (*t*=2.236, *df*=17, and *p*=0.0390) and TNBC subtypes (*t*=2.929, *df*=13, and *p*=0.0117). However, the expression of ADK-L in the Her2 positive subtypes was not significantly different from paired NAT controls (*t*=1.897, *df*=11, and *p*=0.0844) (Figure 1C). In contrast, the expression levels of ADK-S was not significantly different in breast cancer tissues compared to that of paired NAT controls for the whole group analysis (paired t-test, *t*=0.1929, *df*=45, *p*= 0.8479) (Figure 1A, 1B); similarly, further analysis based on subtypes of breast cancer did not reveal different ADK-S expression levels between cancer versus NAT controls (Figure 1C). Together, ADK-L expression significantly increased in breast cancer tissue, particularly in positive luminal A and B and TNBC subtypes, whereas no significant change of ADK-S was detected in breast cancer versus control.

### CRISPR/Cas9 manipulation of ADK isoforms in breast cancer cells

**Figure 2:**
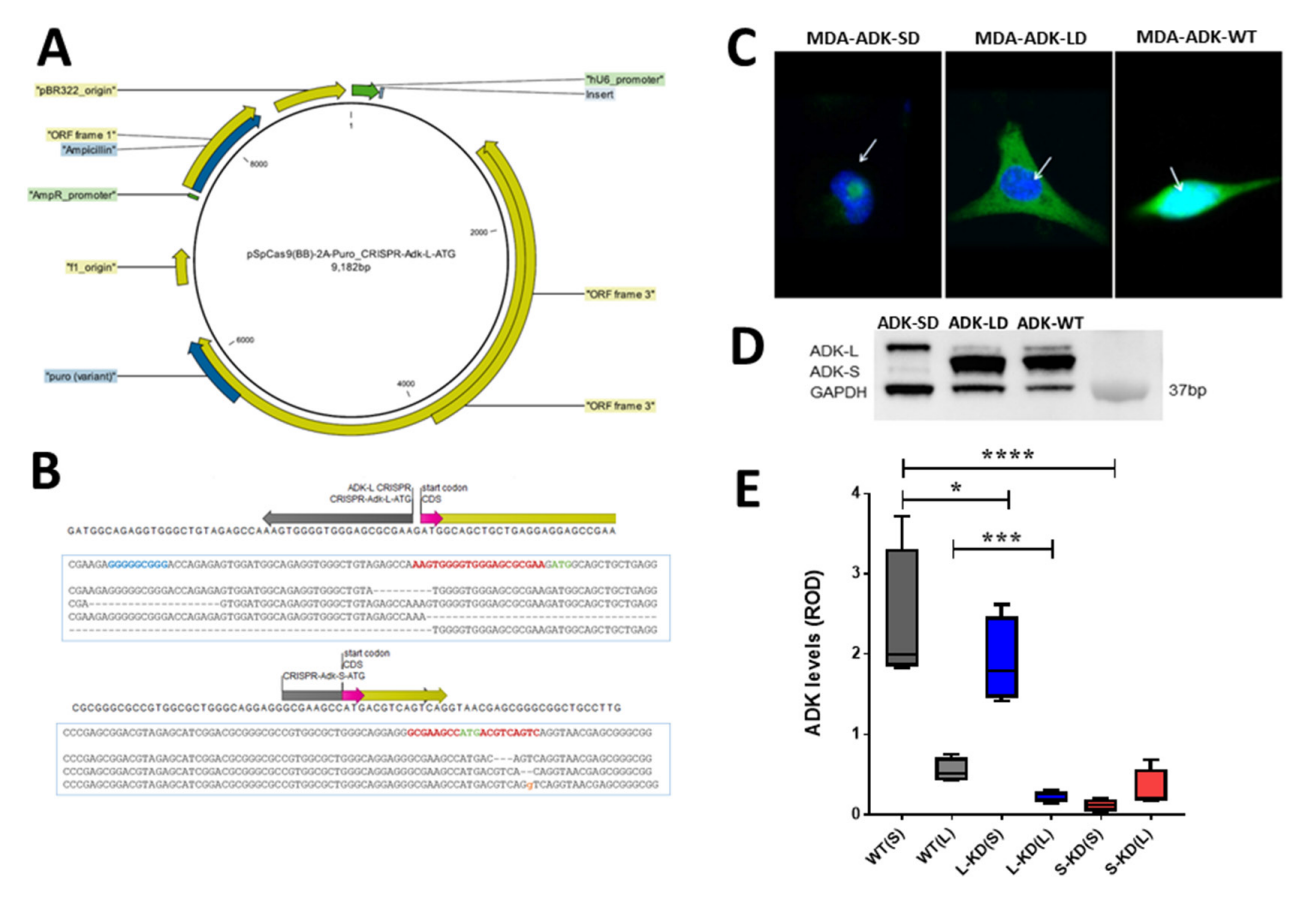
CRISPR/Cas9 manipulation of ADK isoforms in breast cancer cells. **(A)** Plasmid map of CRISPR/Cas9 with a gray insert targeting either ADK-L or ADK–S start codon. **(B)** The schematic figure of the *Adk* gene is shown: ADK-L and ADK-S start codons (in pink), ADK-S CRISPR binding region (in grey), and coding sequences (in yellow) are annotated. **(C)** Representative confocal microscopy images showing subcellular distribution of ADK (in green) expression with DAPI (in blue) with knockdown of ADK-S (left, MDA-ADK-SD), ADK-L (middle, MDA-ADK-LD), or non-modified MDA-MB-231 (right, MDA-ADK-WT) cells. **(D)** Representative image of ADK Western blot and quantitative analysis of expression of ADK isoforms in breast cancer cells with knockdown of ADK-L (MDA-ADK-LD), ADK-S (MDA-ADK-SD), or MDA-ADK-WT cells. **(E)** Quantitative analysis of ADK Western blot showing expression changes of ADK isoforms in MDA-ADK-LD and MDA-ADK-SD cells. * p<0.05; *** p<0.001; **** p<0.0001.

To further evaluate the role of ADK isoforms in breast cancer cellular pathology, we established an *in vitro* model with CRISPR/Cas9 mediated manipulation of ADK in breast cancer (Figure 2A). The distinct start codon of ADK-L and ADK-S isoforms in breast cancer MDA-MD-231 cells were separately targeted with the CRISPR/Cas9 system (Figure 2B). Figure 2C shows ICC visualization of ADK-L or ADK-S knockdown occurred locally in either the nuclear or cytosolic compartment of cells, respectively. The CRISPR/Cas9-mediated knockdown of ADK-L or ADK-S led to correspondingly decreased expressions of ADK-L or ADK-S in MDA-MB-231. To avoid a heterogeneity effect in the CRISPR/Cas9 manipulated cell population, we further focused on two selected single-cell mutant clones to precisely dissect separate ADK isoform-mediated effects on cell proliferation. The decrease of ADK-L and ADK-S in CRISPR/Cas9 transfected cancer cells was evidenced by Western blot assay of MDA-ADK-LD and MDA-ADK-SD cells (one-way ANOVA, for ADK-L, *F*_(2,9)_ =13.51, *p*=0.0019; and for ADK-S, *F*_(2,9)_ =63.31, *p*<0.0001) (Figure 2D). Specifically, (i) the ADK-L level in MDA-ADK-LD cells significantly reduced to 39% of the basal ADK-L level in MDA-ADK-WT cells (p=0.0008, Fisher’s LSD comparison test); and (ii) the ADK-S level in MDA-ADK-SD cells significantly reduced to 5.8% of the basal ADK-S level in MDA-ADK-WT cells (p<0.0001, Fisher’s LSD comparison test). Noticeably, MDA-ADK-SD cells also have a reduction of ADK-L to 53% of the basal ADK-L level (p=0.004, Fisher’s LSD comparison test) (Figure 2E).

### ADK downregulation suppressed cancer cell proliferation and viability

**Figure 3:**
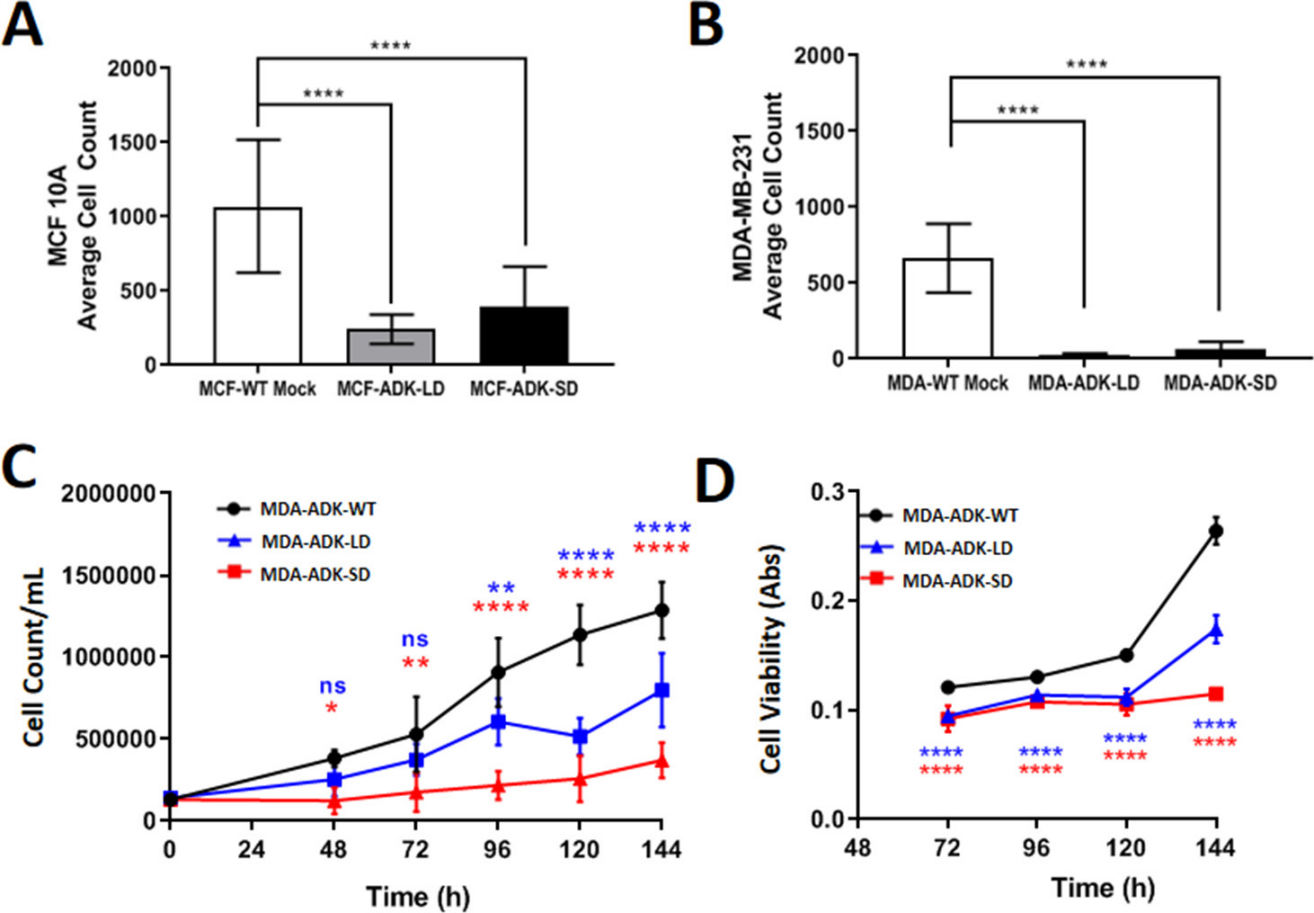
ADK manipulations in breast cancer cells and its effect on growth and viability. **(A)** MCF 10A (non-cancerous breast cell line) and **(B)** MDA-MB-231 (breast cancer cell line) cell proliferation comparison after ADK-L and ADK-S CRISPR transfection. 1 million cells seeded 24h prior to transfection in 6-well plates; cell count performed after Ki-67 staining (n=20). **(C)** Growth curve of cultured MDA-MB-231 cell lines as quantified by Trypan Blue cell counting. Both ADK-L and ADK-S knockdown mutant cells have longer doubling times than unmodified MDA-ADK-WT cells **(D)** Cell viability over time was quantified by MTT assay. As measured formazan absorbance is directly proportional to the number of viable cells, it can be seen that viability is decreased in both modified cell lines.

Using our established CRISPR/Cas9 approach of targeting the start codon of each ADK isoform, we further evaluated the effect of ADK-L or ADK-S knockdown in MDA-MB-231 breast cancer cell line on cell proliferation and viability. Cell proliferation data showed that ADK-L and ADK-S knockdown led to a reduced proliferation rate in both MDA-MB-231 (i.e., MDA-AKD-LD and MDA-ADK-SD) and MCF 10A (i.e., MCF-ADK-LD and MCF-ADK-SD) cells (Figure 3A, 3B). This suppression effect was found to be stronger in the breast cancer MDA-MB-231 cells than in the corresponding MCF 10A cells with knockdown of ADK-L or ADK-S (normalized to mock transfection, one-way ANOVA and Tukey’s Multiple Comparison Test, *F*_(2,3)_ =25.37, *p*=0.0132). Our data showed that a significant decrease in cell growth rate in both of the engineered MDA-ADK-LD and MDA-ADK-SD cells. Also, the doubling time of MDA-ADK-LD and MDA-ADK-SD slowed drastically versus MDA-WT cell (F _(2,68)_ =82.8, p<0.0001; Tukey’s Multiple Comparison Test) (Exponential growth analysis (Y=Y0*e^k*X^), Doubling times (in hours): MDA-WT: 41.17, MDA-ADK-LD: 54.88, MDA-ADK-SD: 108.5) (Figure 3C). In addition, the cell viability showed significant drops in MDA-ADK-LD and MDA-ADK-SD cells (Exponential growth analysis (Y=Y0*e^k*X^), Doubling times (in hours): MDA-WT: 53.93, MDA-ADK-LD: 80.63, MDA-ADK-SD: 268.3) (Figure 3D). These results indicate that ADK long and short isoforms are needed for cell growth, whereas the knockdown of ADK-L or ADK-S suppressed cellular proliferation in MDA-MB-231 breast cancer cells.

### Knockdown of ADK isoforms suppressed migration and invasion of breast cancer cells

**Figure 4:**
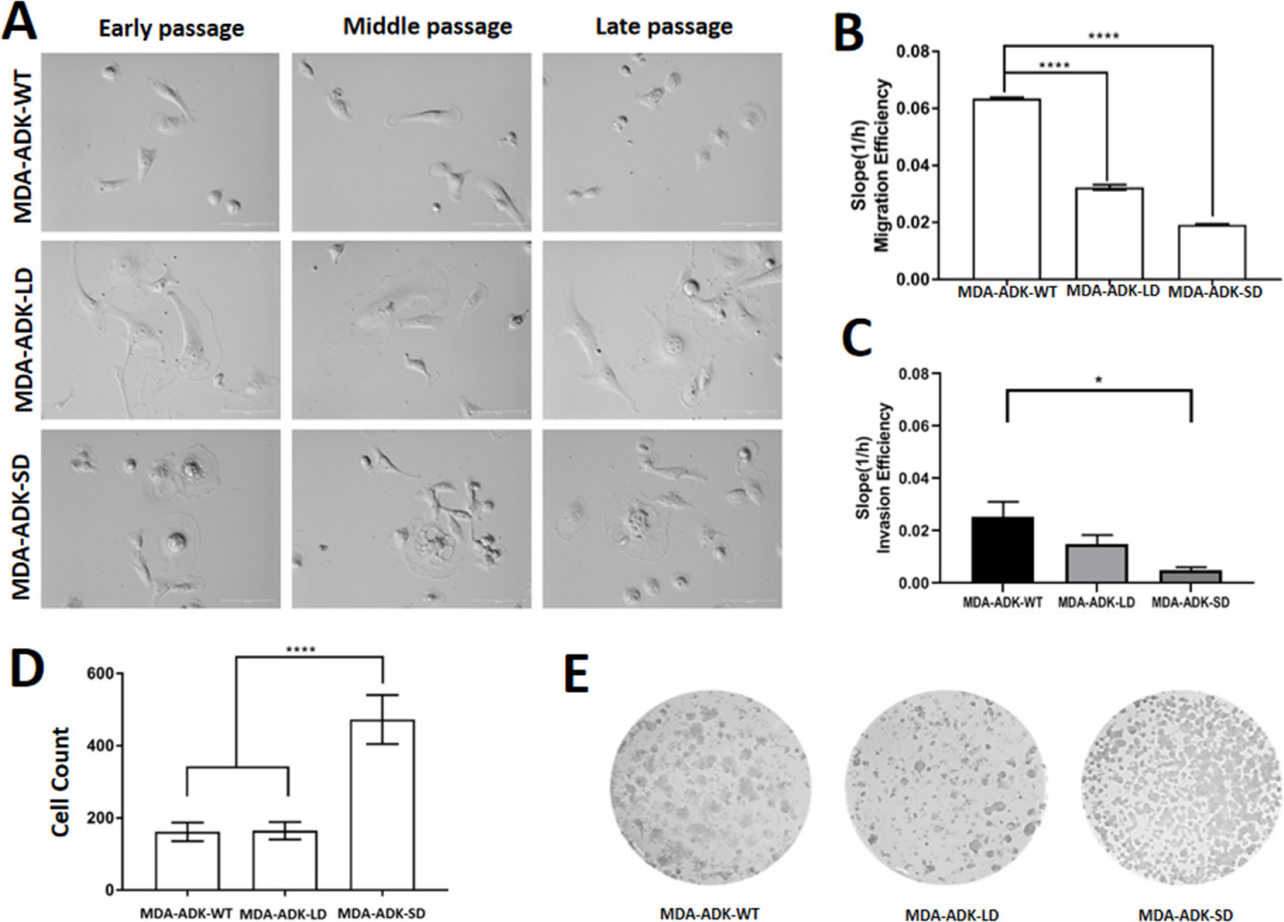
Manipulation of ADK isoforms affects morphology, migration, and invasion of cultured MDA-MB-231 cells. **(A)** Cell morphology of MDA-MB-231 cells with manipulation of either ADK-L knockdown (MBA-ADK-LD), ADK-S knockdown (MBA-ADK-SD), or without ADK changes (MBA-ADK-WT), in their early, middle, and late passages. The MBA-ADK-LD cells look bigger and flatter, while a necrotic morphology was observed on the MBA-AKD-SD cells. **(B)** Both knockdowns of ADK-L (MDA-ADK-LD) and ADK-S (MDA-ADK-SD) reduced migration of cultured MDA-MB-231 cells without ADK modification (MDA-ADK-WT). **(C)** Knockdown of ADK-S (MDA-ADK-SD) reduced the invasion efficiency of MDA-MB-231 (MDA-ADK-WT) cells **(D)** Anchorage-independent colony formation assay and **(E)** representative images of cell colonies of MDA-MB-231 cells with modification of ADK isoforms. ** p<0.01; *** p<0.001; **** p<0.0001.

We observed overt morphological changes in MDA-MB-231 (i.e., MDA-ADK-WT) cells with knockdown of ADK isoforms. For instance, the MDA-ADK-LD mutant cell showed an enlarged and flat cell morphology (Figure 4A). MDA-ADK-SD cells showed apoptosis-like morphologic phenotypes, such as shrinkage of the cell and the nucleus; importantly, the MDA-ADK-SD mutant cells would stop dividing in their late passages whereas MDA-WT cells continue dividing. Further qualitative analysis of cell migration data showed that MDA-ADK-LD cells have significantly decreased migration ability compared to MDA-WT cells (one-way ANOVA, *F*_(2,15)_=94.67, *p*<0.0001) (Figure 4B). In addition, the invasion ability of both MDA-ADK-LD and MDA-ADK-SD cells showed a reduction trend compared to MDA-WT cells in a small-scale trial set, though significance was only observed in MDA-ADK-SD cells (one-way ANOVA, *F*_(2,7)_=5.065, *p*<0.0436) (Figure 4C). Lastly, anchorage independence colony formation assays showed that MDA-ADK-SD cells formed more colonies in soft agar compared to MDA-ADK-LD or MDA-WT cells (*F*_(2,21)_=130.8, *p*< 0.0001) (Figure 4D, 4E).

### Manipulation of ADK isoforms altered gene expression profiles in breast cancer cells

**Figure 5:**
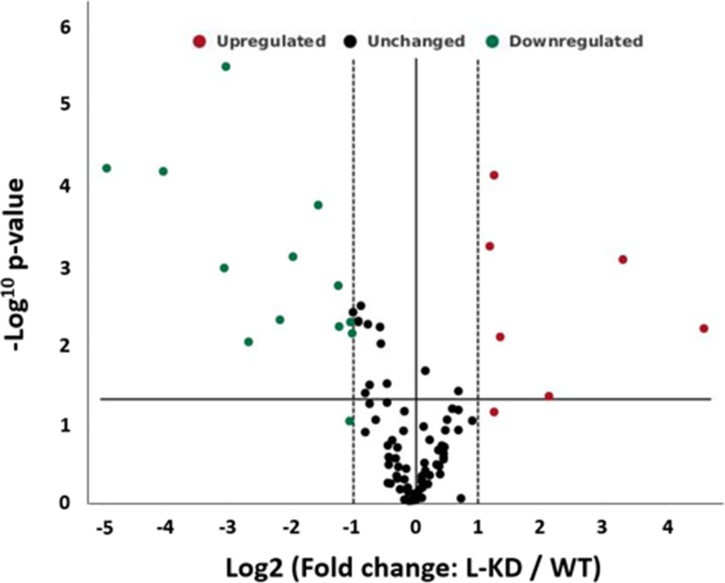
Knockdown of ADK-L affected gene expression of MDA-MB-231 cancer cells. Volcano plot displaying statistical significance versus fold-change of expression on the y- and x-axes, respectively. Of the 89 genes assayed for expression change, those significantly downregulated are shown in green; those upregulated shown in red. Fold regulation threshold of 2, *p* value of 0.05.

While ADK-S manipulation controls extracellular availability of adenosine and activation of adenosine receptors, we focused on mechanistically understanding the role of ADK-L on cancer pathology. To evaluate the possible mechanisms and genes that contribute to cellular phenotypic changes of ADK modified MBA-MB-231 breast cancer cells, we explored the gene expression profiles using an RT2 profiler PCR array and compared 84 genes between cultured MDA-MB-231 cells with or without ADK-L knockdown (i.e., MDA-ADK-LD vs MDA-WT) (Figure 5). The expression data showed that knockdown of ADK-L induced a distinct gene expression pattern, which particularly led changes in three aspects, i.e., matrix metalloproteinase, cell cyclin protein, and adhesive glycoprotein. Specifically, MDA-ADK-LD cells have (i) a significantly downregulated expression of cyclin D2 gene (CCND2, -30.76-fold, vs MDA-WT cell), indicating the role of ADK-L on tumorigenesis and mitogenesis; (ii) an upregulated expression of matrix metalloproteinase genes (ADAM23, 9.93-fold; and MMP9, 24.58-fold), suggesting a role of ADK-L on metastasis and migration of MDA-ADK-LD cell; and (iii) a downregulation of an adhesive glycoprotein gene (THBS1, -8.28-fold), which link to cell-cell and cell-matrix interactions (Table 1). All the above altered gene expression changes suggest that ADK-L is tightly linked to cancer cell pathology in the aspects of proliferation and mitogenesis, as well as tumor-associated tissue remodeling and invasion.

**Table 1:** ADK-L knockdown induced expression changes in MDA-MB-231 cancer cell line

Gene symbol	Gene name	Fold change	p-value
ABCG2	ATP binding cassette subfamily G member 2	-2.36	0.001864
ADAM23	ADAM metallopeptidase domain 23	9.93	0.000875
CCNA1	Cyclin A1	2.57	0.008173
CCND2	Cyclin D2	-30.76	0.000063
CSF1	Colony stimulating factor 1	-2.94	0.000182
CST6	Cystatin E/M	-16.32	0.000069
FOXA1	Forkhead box A1	-2.01	0.007372
GRB7	Growth factor receptor bound protein 7	2.28	0.000597
ID1	Inhibitor of DNA binding 1	-6.37	0.009457
IGFBP3	Insulin like growth factor binding protein 3	-2.33	0.006068
MMP9	Matrix metallopeptidase 9	24.58	0.006421
MUC1	Mucin 1, cell surface associated	-3.90	0.000806
PYCARD	PYD and CARD domain containing	-2.05	0.005339
RARB	Retinoic acid receptor beta	4.39	0.045406
SFN	Stratifin	2.39	0.000077
SLIT2	Slit guidance ligand 2	-4.47	0.005002
TFF3	Trefoil factor 3	-8.33	0.001119
THBS1	Thrombospondin 1	-8.13	0.000003

Notes: Fold Regulation comparison and p-value of RT2 profiler PCR Array MDA-ADK-LD vs MDA-MB-231 cancer cell line. Fold change/regulation was calculated using the delta-delta C_T_ method, in which delta C_T_ is calculated between the gene of interest and an average of reference genes, followed by delta-delta C_T_ calculations [delta C_T (Test Group)_-delta C_T (Control Group)_]. Fold-change values greater than one indicates a positive- or an up-regulation, and the fold-regulation is equal to the fold-change. Fold-change values less than one indicate a negative or down-regulation, and the fold-regulation is the negative inverse of the fold-change. The p values are calculated based on a Student’s t-test of the replicate 2^(-Delta C_T_) values for each gene in the control group and treatment groups.

## DISCUSSION

Interest in ADK has increased regarding its role in modulating the adenosinergic pathway in cancer [4–8, 25]. However, the role of the two different isoforms of ADK in tumorigenesis, and specifically with the progression of breast cancer, remains unclear. In this present study, we explored expression profiles of ADK isoforms in tumor tissues of 46 breast cancer patients and breast cancer cell lines. Furthermore, we established a CRISPR/Cas9 system to selectively modify each ADK isoform in cultured MDA-MB-231 breast cancer cells and scrutinized the contributions of different ADK isoforms on cancer cell pathology. Our findings demonstrated that (i) expression of ADK-L is significantly increased in clinical breast cancer tissues, especially in the patient cases with luminal A- and B-positive and TNBC subtypes; (ii) CRISPR/Cas9-mediated downregulation of ADK isoforms, led to a suppression of cellular proliferation, division, and migration of cultured breast cancer cells; and, of particular note, (iii) knockdown of ADK-L in cultured MDA-MB-231 cancer cells (i.e., MDA-ADK-LD) upregulated the expression of matrix metalloproteinase genes (ADAM23 and MMP9) and downregulated expression of the cyclin D2 gene and an adhesive glycoprotein gene (CCND2 and THBS1). The present study, for the first time, provides experimental evidence to mechanistically dissect the differential roles of two ADK isoforms in cancer pathology.

### ADK isoforms act differently in tumorigenesis and cancer development

A well-accepted notion is that ADK-S (generally named as ADK in previous publications) regulates intracellular adenosine processing and extracellular availability of adenosine for its activation of adenosine receptors (e.g., adenosine A_1_, A_2A_, A_2B_, and A_3_ subtype receptors); as related to cancer, these actions affect the angiogenesis, cell proliferation, apoptosis, and tumor immune response. On the other hand, while having been characterized and identified for a decade, the action of nuclear ADK-L is still largely unknown and underexplored [16]. While we revealed in this study that ADK-L expression significantly increased in breast cancer, the ADK-S expression, in contrast, was devoid of significant changes in expression between cancer and normal breast tissue. Due to the observed different expression profiles of ADK-L and ADK-S, as well as their different molecular features and distinguished subcellular location, it is natural to suspect that the two ADK isoforms may act via different mechanisms in cell biology and cancer pathology. Indeed, a newly proposed mechanism of isoform switching in dysfunctional cells was evidenced in a recent human cancer study from twelve solid cancer types [26], which may also apply to ADK isoforms on tumorigenesis.

Triple negative breast cancer (TNBC) is the most aggressive subtype and has the poorest prognosis compared to other types of breast cancer. In our *in vitro* study using the selected TNBC cell line, MDA-MB-231, we generated CRISPR/Cas9-mediated, isoform-selective ADK knockdown in breast cancer cell lines and further evaluated the role of these two ADK isoforms on phenotypic changes of MDA-MB-231 cells with engineered manipulation of ADK-L or ADK-S. Indeed, the manipulation of ADK-L or ADK-S *per se* can lead to cellular changes in proliferation, differentiation, and metastasis. Importantly, our findings indicate that the nuclear ADK-L plays a crucial role in mitogenesis and cell differentiation – two major nuclear activities in cancer pathogenesis [27, 28], as well as regulating tumor tissue remodeling and invasion [29]. We provide experimental evidence that supports a direct nuclear effect of ADK-L via the mediation of gene expression (see discussion in the next section). Recent findings from our lab and peers also suggest that ADK-L potentially participates in epigenetic modifications [19], which may contribute to the nuclear effects of ADK-L. This effect could be independent from the ADK-S-centric adenosine receptor activation. While our data suggest that targeting ADK-L might be of therapeutic interest, this notion poses a question arising from epidemiological studies showing that caffeine intake (thus blocking adenosine receptors) is related to a decline in the incidence and evolution of some of the different types of breast cancer [30–32]. These findings might suggest an alternative interpretation of the mechanisms underlying the role of ADK. Therefore, further study is warranted to dissect more specifically the individual actions of these two ADK isoforms.

### ADK-L affects the expression of genes linked to cancer cell proliferation and invasion

Proliferation and migration of cancer cells are two of the major targeting aspects of cancer treatment. After describing the altered cellular proliferation and migration behaviors of MDA-MB-231 cells with knockdown of ADK isoforms, we further demonstrated that ADK-L strongly affects multiple gene expressions - including *CCND2, MMP9, ADAM23, THBS1,* and *CST6* - that are relevant to cancer cell proliferation and migration/invasion (Figure 5 and Table 1). Firstly, cyclin D2 (CCND2) is a member of the D-type cyclins, which plays a pivotal role in cell cycle regulation, differentiation, and malignant transformation. While the role of CCND2 is still controversial in a different type of tumors, high-level expression of CCND2 was observed in testicular and ovarian tumors [33, 34], and aberrant promoter methylation status of CCND2 was shown in breast cancer tissues [35, 36]. CCND2 interacts with the phosphorylation of the tumor-suppressing retinoblastoma protein Rb [37], and it is also a target in TNBC [38], as the CpG loci are differentially methylated in various breast cancer tumor subtypes [39]. Thus, knockdown of ADK-L-decreased CCND2 expression may affect CCND2-related actions on cell cycle regulation, cancer cell growth inhibition, and migration ability [36]. This effect might not only occur in breast cancer but also could be a general feature of cells with dysregulated cell cycle. Secondly, metalloprotease and adhesion molecules, MMP9 and ADAM23, are involved in the breakdown of the extracellular matrix in physiological processes such as embryonic development, reproduction, and tissue remodeling [40]; they also play an important role in metastasis of cancer [41]. Studies have shown that a loss of expression of ADAM23 gene and its correlation with promoter methylation has been frequently reported in breast cancer, brain cancer, and pancreatic cancer [42, 43]. Particularly, ADAM23 suppresses cancer cell progression through interaction with αvβ3 integrin [44]; the upregulation of ADAM23 in this study due to ADK-L knockdown may facilitate this effect. Similarly, MMP-9 participates in tumor-associated tissue remodeling [45]. The MMP-9 expression varies according to the molecular subtypes of breast cancer [46]. Interestingly, while MMP-9 has a suggested link to malignant progression and metastasis of TNBC [47] and histological breast cancer grades [46], inflammation induced by MMP-9 can enhance tumor regression of experimental breast cancer [48]. These discrepant effects of MMP9 seem to be consistent with its diverse expression pattern across subtypes of breast cancers, which needs further exploration. Further, while the effects of ADK-L might be unrelated to adenosine receptor function, ADK knockdown-mediated expression changes on proteins that are crucially involved in cancer pathology warrant further investigation for insights on potential mechanisms as a novel therapeutic target.

### Limitation and remarks

While we, for the first time, revealed the expression profile of ADK isoforms in breast cancer and several tumor subtypes, the present study has limitations, particularly the relatively small patient sample size and lack of direct immunohistological evaluation of the subcellular distribution of ADK isoforms in cancer tissues. We acknowledge that the limited sample size also compromised the statistic power necessary to specifically evaluate expression levels of ADK isoforms according to clinico-pathological features. To further explore the profile of ADK isoforms in breast cancer, we have investigated the ADK expression profiles in several established breast cancer cell lines, including MDA-MB-231, and MCF7. We observed different expression levels of ADK isoforms among these cancer cell lines as well as between non-cancer cell lines, such as MCF 10A and 184A1 (data not shown), which might be attributed to the heterogeneity of these cell lines. Additionally, future investigations are necessary to determine how modification of the methylated DNA profile in ADK-L differs from that of ADK-S. Therefore, while we provide promising findings from the manipulation of ADK isoforms in cellular behaviors of cancer cells, it is still premature to draw any firm conclusions regarding ADK isoforms as a specific biomarker in breast cancer. Further investigations with larger-sized patient studies and more extensive *in vitro* experiments involving additional breast cancer cell lines are, therefore, warranted.

Nevertheless, based on our findings here, we concluded that ADK-L inhibition could be a potentially viable therapeutic approach for at least certain subtypes of breast cancer (i.e. luminal A and luminal B, and TNBC). Further studies should be advised that while further dissection of the role of ADK isoforms individually, we must consider the possibility of a joint- and/or compensatory effect of knockdown ADK-S and ADK-L. Therefore, we believe that ADK targeting has more potential as a cancer treatment target once the causal manipulatory effects of ADK have been confirmed with future *in vivo* studies.

## MATERIALS AND METHODS

### Tumor specimens and cell lines

A total of 46 breast cancer specimens (luminal A, luminal B, Her2+ and triple negative breast cancer) from female patients were involved in this study. The cancer tumor specimens and normal tissues adjacent to the tumor (NAT) were collected by Legacy Tumor Bank (Legacy Health, Portland, OR USA). The pathological diagnosis data were deidentified and used for subcategorization in this study.

All the cell and virus studies were conducted with protocols approved by the Institutional Biosafety Committee of the Legacy Research Institute and in accordance with the principles outlined by the NIH. The following cell lines used in this study were purchased from the American Type Culture Collection (AATC, Manassas, VA): MDA-MB-231 (HTB-26), MCF7 (HTB-22), MCF 10A (CRL-10317). The breast cancer MDA-MB-231 cell line (called MDA-WT cell in the following text) was used to investigate the phenotypical changes after introducing engineered CRISPR/Cas9 modification of ADK isoforms (details see next section).

### CRISPR/Cas9 plasmid constructions

To manipulate the ADK long or short isoforms in breast cancer cells, two sgRNA inserts were designed to target ADK-L and ADK-S (20 nt) start codons, which were cloned into pSpCas9(BB)-2A-GFP (PX458) plasmid (Addgene plasmid # 48138, Watertown, MA) and pSpCas9(BB)-2A-Puro (PX459) V2.0 plasmid (Addgene plasmid # 62988, Watertown, MA) using BbsI restriction enzyme [49] – both above plasmids were gift from Dr. Feng Zhang (Addgene, Watertown, MA, USA). The pSpCas9(BB)-2A-GFP and pSpCas9(BB)-2A-Puro plasmids were designed to express sgRNA targeting human ADK-L or ADK-S start codons, scaffold RNA (U6 promoter), and Cas9 endonuclease (Cbh promoter) [49] in mammalian cells (Figure 2A, 2B).

### Screening and identification of genetically engineered single cell clones

To screen ADK-L and ADK-S mutated MDA-MB-231 cell pools after CRISPR/Cas9 transfection, the cultured cell pools were diluted in 96-well plates to isolate single clones as per Addgene protocol (https://www.addgene.org/protocols/limiting-dilution). Briefly, cells isolated from a stable cell pool were homogenized and serially diluted to a concentration of 5 cells/mL. A 100uL of this solution was seeded to each well of a 96-well plate for an average density of 0.5 cells/well. Single cells were left to grow into colonies and transferred before reaching confluency as independent monoclonal lines. Limiting dilution was performed twice for each line to validate homogeneity. Cultured single cell clones were then harvested for screening for the ADK isoform mutation using restriction fragment length polymorphism (RFLP) and Western blot assay of ADK. Several single cell clones with the desired mutation of ADK isoforms were detected (data not shown), from which two single cell clones, one mutant clone with ADK-L knockdown (MDA-ADK-LD), another mutant clone with ADK-S knockdown (MDA-ADK-SD), were selected and used in this study (Figure 2C).

### Western blot assay

To quantify expression changes of ADK isoforms in the patient specimens and cultured cells, Western blot assays were conducted as described [50]. Briefly, patient specimens and harvested cultured cells were homogenized and digested using RIPA buffer (10 mM Tris-Cl, pH 8.0, 1 mM EDTA, 1% Triton X-100, 0.1% sodium deoxycholate, 0.1% SDS, 140 mM NaCl, 1 mM PMSF) to prepare extracts. Extracts were standardized to 40 μg protein per lane and electrophoresed in a 10% Tris-glycine gel. After transfer, membranes were incubated in primary antibody anti-ADK antibody (#A304-280A, 1:5,000; Bethyl lab, Montgomery, TX) followed by incubation with peroxidase-conjugated anti-rabbit antibody (#7074, 1:8,000, Cell Signaling, Boston, MA). The anti-ADK antibody was used to detect both isoforms of ADK distinguished by their molecular weights. To normalize ADK immunoreactivity to protein loading, a mouse monoclonal anti-α-tubulin antibody (# sc-8035, 1:5,000; Santa Cruz, Santa Cruz, CA, USA) or GAPDH antibody (1:5,000; #14C10, Cell Signaling, Boston, MA) was used to reprobe the same blot and the OD ratio of ADK to α-tubulin or GAPDH was calculated. The intensity of immunoblots was quantified using Image Lab software (BioRad, Herculer, California, USA).

### Immunocytochemistry

Immunocytochemistry of ADK was conducted to evaluate the expression pattern of ADK isoforms of our engineered MDA-MB-231 breast cancer cells with ADK-L knockdown (MDA-ADK-LD) or ADK-S knockdown (MDA-ADK-SD). Cells were seeded in 6-well plates on sterile coverslips to 50-60% confluence, washed with ice-cold TBS, and fixed with 4% (v/v) paraformaldehyde for 20 minutes. The fixed cells were washed with TBS and permeabilized with TBS-T. Antigen retrieval was performed with 2N HCl which was neutralized with Tris-base and rinsed in TBS-T. Subsequently, the fixed cells were blocked in blocking solution (Goat Blocking Buffer TBS with BSA and Triton) for 1 h at room temperature. After blocking, the fixed cells were incubated in primary antibody anti-ADK (#A304-280A, 1:1000; Bethyl Lab, Montgomery, TX) or anti-Ki-67 (#sc-23900; 1:500; Santa Cruz, Burlingame, CA) overnight, then incubated in secondary antibody solution for 90 minutes (#A-11034, 1:350; Alexa488 Life Tech, Waltham, MA). Coverslips were sealed with Vectashield Mounting Medium with DAPI (#H-1200; Vector Labs, Burlingame, CA) and imaged using a Leica inverted confocal microscope. Control staining without primary or secondary controls was included for all cell types in our ICC staining.

### Cell proliferation and viability assay

Cell proliferation was quantified by Trypan Blue cell counting [51]; after seeding, cell counting was performed every 24 h for several consecutive days. MTT assay was performed to evaluate the cell viability of WT cells versus mutant cells using MTT assay Kit (Cell Proliferation Kit I, Sigma Aldrich, St. Louis, MO) according to the manufacturer’s protocol.

### Gene expression assay

The expression of specific genes in MDA-MB-231 breast cancer cells with ADK-L knockdown (MDA-ADK-LD) was compared with non-modified MDA-MB-231 cells using RT2 profiler PCR array kit. Briefly, RNA was extracted using an RNeasy kit (Qiagen, #74134), with a total of 0.5 μg of RNA used for the amplification of each sample. cDNA was created as per the protocol of RT2 First Strand Kit (Qiagen, #330404) and amplified within the RT2 Profiler PCR Array for Human Breast Cancer (Qiagen, #330231, PAHS-131Z) with RT2 SYBR Green Mastermix (Qiagen, #330500). Two biological replicates of matching passage age of each MDA-MB-231-ADK-L-KD and MDA-MB-231 WT cells were isolated and analyzed with two technical replicates for a total of 8 complete assay plates. Thresholding values were normalized across all plates as per the RT2 Profiler PCR Array protocol.

### Assessment of cell migration and invasion

The xCELLigence RTCA DP instrument and migration assay were employed to assess the ability of cellular migration and invasion according to publications [52] with modifications. Briefly, 100 μl of cell suspension (30,000 cells per well) was added to the upper chamber of the CIM-Plate 16 transwells. The lower chamber of the transwell contained Leibovitz’s L-15 with 10% FBS (as the chemoattractant) or medium without FBS (serum-free medium: SFM) for negative control. CIM-Plate 16s were placed in xCELLigence RTCA DP instrument and the migration assay was run for 24h. The migration efficiency of the cells was compared with each other after the run was complete. Invasion assay was performed by applying 50 μl Vitrogel 3D-RGD (The Well Bioscience, Newark, USA) on the microporous membrane of the CIM-Plate 16. Cells were expected to invade through the membrane to reach the nutrition (10% FBS medium) in the lower chamber. Invading cancer cells were sensed by microelectrodes and invasion assay was further analyzed via RTCA DP software.

### Anchorage independence assay

To evaluate the changes of ADK modification on cellular ability of metastasis, anchorage independence assays were performed on cultured MDA-MB-231 cells with or without knockdown of ADK isoforms. Briefly, MDA-ADK-LD and MDA-ADK-SD cell lines were cultured in soft agar in 6-well plates for 21 days. Petri dishes had 2 layers of agar; base layer (1% agar) and the top layer (0.7% agar) that contained the cells. Agar layers were prepared with low-gelling-temperature agarose (A9045, Sigma, St. Louis, MO, USA) and Leibovitz’s L-15 powder medium without Phenol red (HIMEDIA, AT204, Mumbai, India). The top layer of each assay contained 5000 cells. 6-well plates were maintained at 37°C in a humidified incubator, and 0.5 ml fresh growth media was added to each plate twice weekly. Colonies were stained with 0.005% crystal violet for 15 minutes, washed twice with PBS, and manually counted.

### Data analysis

Statistical analysis was performed with ANOVA, t-test, or non-linear regression using GraphPad-Prism8 software. A *p*< 0.05 was accepted as statistical significance. Where applicable, values expressed as mean ± SEM.
